# Factor structure and longitudinal changes in bilinguals’ oral narratives production: role of language exposure, language-domain proficiency, and transfer

**DOI:** 10.1017/s0142716425000050

**Published:** 2025-02-27

**Authors:** Joseph Hin Yan Lam, Molly Ann Leachman, Cecilia Del Carmen Perez, Amy S. Pratt, Elizabeth D. Peña, Lisa M. Bedore, Ronald B. Gillam

**Affiliations:** 1 School of Education, University of California, Irvine, CA, USA; 2 Caruso Department of Otolaryngology-Head and Neck Surgery, Keck School of Medicine, University of Southern California, Los Angeles, CA, USA,; 3 Department of Communication Sciences & Disorders, University of Cincinnati, Cincinnati, OH, USA,; 4 Department of Communication Sciences and Disorders, Temple University, Philadelphia, PA, USA; 5 Communicative Disorders and Deaf Education, Utah State University, Logan, UT, USA

**Keywords:** Bilingualism, cross-language transfer, dynamic systems theory, language exposure, narrative

## Abstract

This paper examined the interaction between narrative performance, language exposure, and standardized measures of morphosyntax and semantics, in bilingual children tested two times, 1 year apart. We aimed to 1) identify the factor structure of oral narrative measures, and 2) examine the direction and strength of the effects of (i) language exposure and (ii) the relationship between language domains and narrative production. A total of 143 Spanish (L1)-English (L2) bilingual children completed a battery of oral narrative and oral language proficiency assessments in Spanish and English at two time points (kindergarten and Grade 1). Factor analyses yielded an identical two-factor structure of bilingual oral narrative measurements, namely productivity (word production) and complexity (sentence structure), in both Spanish and English across the two time points. Cross-lagged analysis showed that narrative production predicted semantics and morphosyntax performance in Spanish and English one year later. Cross-language transfer from L1 to L2 on the complexity of narrative was noted. Language exposure predicted only Spanish narrative production, but not English. These results suggest within- and cross-language transfer, highlighting the importance of L1 language development. In addition, current findings highlight the importance of language exposure for L1 in early school-age children.

Narrative development during early childhood for bilingual children is complex, evidenced by the ways that many of these children use their skills across languages to tell stories. The language development of heritage bilinguals is affected by the quantity, variability, and type of language input they receive. For example, studies have shown that language experience may lead to different outcomes in grammatical acquisition and language use (Albudoor et al., 2024; Polinsky & Scontras, 2020). It is widely understood that early narrative development predicts later literacy skills because oral language skills can facilitate understanding of the meaning of text (Gardner-Neblett & Iruka, 2015; Storch & Whitehurst, 2002). Specifically, development of narrative skills can help children connect oral discourse and written texts. One common way to measure narrative production is through the assessment of narrative microstructure, as it provides a way of understanding a child’s lexical-semantic and grammatical knowledge (Fiestas & Peña, 2004; Iluz-Cohen & Walters, 2012). Narrative microstructure refers to the linguistic elements of oral narration where one must select lexical items and morphosyntactic structures to tell the varying episodes of a story (Squires et al., 2014). However, the longitudinal relationship between language domains (e.g., semantics and morphosyntax) and narrative production in bilinguals remains unclear. Thus, the current study, with reference to DeBot et al. (2007)’s Dynamic Systems Theory, aimed to examine the factor structure of bilingual narrative microstructure and the longitudinal relationship between language exposure, semantics, morphosyntax, and narrative production in Spanish-English bilingual children.

## Narrative developmental milestones and micro-structure measures

According to the comprehensive language approach in typically and atypically developing children, narrative-based developmental milestones related to early school years are important because they are associated with emergent literacy skills (Gardner-Neblett & Iruka, 2015; Kaderavek & Sulzby, 2000; Storch & Whitehurst, 2002; Wellman et al., 2011). In preschool, children have not yet developed the ability to use complex grammatical forms or vocabulary in their narratives (Berman, 1998). However, by the time they enter school, they begin to incorporate microstructural elements that make their stories more complex. For instance, by school age, children use adverbs, elaborate noun phrases, and produce cohesive devices (e.g., coordinating and subordinating conjunctions (Curenton & Justice, 2004; Eisenberg et al., 2008; Gillam & Gillam, 2016), which together integrate microstructural elements of narratives (Gillam et al., 2012; Squires et al., 2014). Thus, age affects narrative development and its underlying factor structure.

Language sampling has been used to measure children’s language performance across several expressive domains of narrative language (Gibson et al., 2018). Previous studies on monolingual narrative microstructure (i.e., the use of linguistic devices at the sentence level to convey meanings) are measured via microproductivity and complexity (Hughes et al., 1997). Productivity quantifies the processes of word and sentence formation. Its common indices include total number of words (TNW), total number of different words, total number of utterances (TNU), and number of grammatical forms (e.g., verbal tense/aspect/voice/inflectional morphology). Indices of sentence complexity include mean length of utterance in words (MLU-w), subordination index (SI), and grammaticality (Miller, 1981; Templin, 1957; Tsimpli et al., 2016). Despite the diverse measurements adopted in various studies in investigating narrative production, it is not clear to what extent these guidelines are generalizable to (Spanish-English) bilingual children given that differing language experiences may impact the extent to which these measures index bilingual development in their two languages. There are no studies investigating the underlying factors of these micro-level narrative measurements in the L1 (first or home language) and L2 (second language) in early school-age bilingual children. Therefore, the first research question was to investigate whether narrative factor structure can be used to explicate the dimensionality of these measures to better understand their underlying constructs and organization of complex language outcomes.

## Within- and cross-language associations over time in bilinguals

Within- and cross-language associations are important to understand bilingual language development. For within-language association, there is a strong relationship between semantics, morphosyntax, and narrative (Kohnert et al., 2010; Simon-Cereijido & Gutiérrez-Clellen, 2009). Cross-language associations are strong for vocabulary but relatively weak in comparison to morphosyntax. As we turn to understand more complex language forms such as narrative, we need a framework that can help us understand how the building blocks of narrative relate to one another and how they are impacted by the child’s language experience. Language models based on dynamic systems theory hold promise in this area. Dynamic systems in development generally explain how multiple inputs interact in open systems to explain qualitative shifts in the resulting outputs or behaviors such as speech or motor development (e.g., Spencer et al., 2011; Thelen, 2013). DeBot’s Dynamic Systems Theory (DST) specifically expands this approach to consider how the small changes between the language constructs in bilingual development (e.g., microstructure elements) and the factors affecting the relationship between these over time (e.g., semantics, discourse, and social factors) (de Bot et al., 2007; de Bot et al., 2013) affect one another. DST describes the relationship between language constructs as *dynamic*, emphasizing that changes in one construct will affect the others via the *butterfly effect*. The butterfly effect suggests that small changes in the initial dynamics may result in large, transformational changes in the outcomes such that later language skills in a complex task such as narrative are qualitatively distinct from the inputs. Furthermore, bilingual developmental trajectories are impacted by the cognitive ecosystem of language learning, including language exposure, language of school instruction, and community practice.

Research demonstrates that semantic and morphosyntactic abilities are positively correlated with each other and/or predict narrative production skills within- and across-language at a single time point in both monolingual and bilingual children (Gardner-Neblett & Iruka, 2015; Sénéchal et al., 2008; Uchikoshi et al., 2018). Changes of this type are in line with DeBot’s suggestion that outcomes are closely related to initial inputs. However, there is limited research examining the longitudinal changes between language-domain proficiency in various language domains and narrative production skills in bilingual children. Uccelli and Páez (2007) investigated vocabulary breadth (i.e., the number of words that a child can name) and narrative production skills from kindergarten to first grade in 24 Spanish-English bilingual children from low socioeconomic backgrounds. Within- and cross-language associations were noted: Spanish vocabulary breadth significantly correlated with English and Spanish narrative skills, and English vocabulary breadth significantly correlated with English narrative skills in kindergarten and first grade. Pham (2016) investigated the vocabulary breadth and narrative production skills in 33 Vietnamese-English bilingual children at four annual intervals. In year one, a significant within-language association was noted between vocabulary breadth and number of different words (NDWs) in both Vietnamese and English, and a significant cross-language association was observed between Vietnamese vocabulary breadth (measured by object and action picture naming) and NDWs in English narratives. However, only the within-language association remained significant after controlling for age and English exposure. In the same study, English vocabulary breadth in year one significantly predicted mean length of utterance (MLU) in English narratives in year four, while there were no significant cross-language predictions (Pham, 2016). These results provided evidence of unidirectional cross-language transfer from L1 to L2 and associations among within-language domains. While suggestive of changes in language dynamics in narrative being associated with context as predicted by DST, a limitation of these studies is that they have small sample sizes and may have high attrition rates (e.g., Pham, 2016: 63.64%) in longitudinal studies. Thus, the relationship between language exposure and the changes in language domains in L1 and L2 still remains unclear. The second research question aimed to explore the relationship between language exposure, and basic language domains (i.e., semantics and morphosyntax) in L1 and L2 complex language domains (narrative) in L1 and L2.

## Changes in narrative skills as related to language exposure

Language environments, age, and cognitive demands may affect narrative outcomes. Extant research indicates that for bilinguals, current exposure to each language predicts their development and thus performance in each language (Bedore et al., 2012, 2016). Current exposure refers to how much time a bilingual spends hearing and using each of their languages at home and in the community throughout a typical week. The relative contribution of cumulative experience (e.g., age of first exposure to English) versus current exposure appears to change with age (Bedore et al., 2012). For instance, research suggests that current exposure plays a more prominent role in children’s language development when children are younger (Bedore et al., 2012). However, as children get older, the contribution of cumulative experience increases and becomes more comparable to current exposure (Bedore et al., 2016).

There are different claims about the relationship between language exposure and bilingual language development. Language growth is hypothesized to be driven by both language exposure and language learning ability in bilingual children. Language exposure is a strong predictor of L1 and L2 language growth and it differentially affects different language domains (Bohman et al., 2010). In addition, language learning ability is also a factor affecting language development. Research has demonstrated that even when exposure is held constant, the lexical-grammatical correlation is stronger within language than across languages (Marchman et al., 2004; Simon-Cereijido & Gutiérrez-Clellen, 2009). The quadratic associations between vocabulary and grammar suggested that higher language learning ability can promote greater language growth (Marchman et al., 2004). Thus, our second research question focused on how current language exposure (input and output), and language-domain proficiency were associated with bilingual narrative production in early school-age children. This can also evaluate the Dynamic Systems Theory by examining the effect of language exposure, which is part of the cognitive ecosystem (de Bot et al., 2007), and changes in complex language outcomes as evidenced in narrative.

## Significance of the study

Investigating the underlying factor structure of narrative production addresses the dimensionality of bilingual narrative production at the micro-level. This also assesses the generalizability of the factor structure suggested by Hughes et al. (1997). Researchers and clinicians can utilize this information to select measurements to evaluate bilingual children’s narrative ability. It also laid the groundwork for the analysis between language domain changes and narrative production changes.

The relationship between (1) within-language-domain proficiency and narrative; and (2) cross-language narrative was examined using cross-lag models. This aligns with the Dynamic Systems Theory, which predicts that the initial state has significant importance on later language outcomes (de Bot et al., 2007). In addition, we can use this approach to examine the transfer of language performance in different contexts. Specifically, language-domain proficiency was measured by elicited responses (e.g., semantic and morphosyntax items on the Bilingual English Spanish Assessment [BESA]) using decontextualized tasks; while narrative production was measured by asking children to tell stories from a wordless picture book which is contextualized, yet cognitive-demanding and relatively complex. The relationship between language domain changes in L1 and L2 has strong theoretical and educational implications. The relationship between language exposure and complex language skills can examine the impact of environment on language outcome (de Bot et al., 2007). Our second research question investigated the longitudinal relationship between language domains and narrative in Spanish and English separately and between English and Spanish narrative production. From an educational perspective, the current study informs educators and practitioners of the potential generalization of the relationship in basic language domains (such as semantics and morphosyntax) on complex language functions in early school age and how language exposure can affect these relationships.

## Research questions

The current study assessed the relationships among common micro-level narrative measurements in L1 and L2 of bilingual early school-age children. The first research question focused on the factor structure between (i) language and (ii) grade of bilingual narrative production. The second research question further explored what factor(s) contribute to the longitudinal relationship in narrative production between kindergarten and Grade 1. Specifically, we asked whether (i) language exposure, (ii) within-language proficiency in semantics and morphosyntax, and (iii) cross-language narrative performance in kindergarten predicted bilinguals’ narrative performance in English and Spanish from kindergarten and Grade 1.

## Method

### Participants

Data for the current study were drawn from an existing dataset of 143 participants in the Diagnostic Markers of Language Impairment project (DM, Peña et al., 2006). Children were Spanish-English bilinguals and completed a battery of language tasks in both of their languages including narrative storytelling. Participants were included in this study if they had completed the narrative and language assessment. The DM study was a three-year longitudinal study that assessed bilingual children annually from pre-kindergarten to Grade 1 from low socioeconomic status neighborhoods. Bilingual oral language development and narrative ability were measured in kindergarten and first grade. Table 1 presents participants’ demographics. Seventeen of the participants had developmental language disorder (DLD) and the decision was made based on the consensus of three experienced speech-language pathologists (for detail, see Peña et al., 2014; Wang et al., 2024). Ethics approval was obtained from the University of Texas at Austin. Informed written consent was obtained from the caregivers of all participating children prior to testing.

### Measures

#### Nonverbal intelligence (Assessed at T1).

The Universal Nonverbal Intelligence Test (UNIT, Bracken & McCallum, 1998) was administered. The UNIT is given non-verbally and children respond non-verbally. The measure yields a standard score based on child age.

#### Language exposure (Assessed at T1 and T2).

The Bilingual Input-Output Survey (BIOS), a parent-reported questionnaire, was administered to evaluate the language exposure of the student (Peña et al., 2018). The BIOS requires caregivers to report the age of acquisition of each language of the child and also the hour-by-hour language exposure (input and output). The percentage of English exposure was calculated according to the manual (Peña et al., 2018). The percentage of English exposure was calculated based on the average of the weekly English input and output, while the percentage of Spanish exposure was 100% minus the percentage of English exposure.

#### Oral language-domain performance (Assessed at T1 and T2).

The semantics and morphosyntax subtests of the BESA (Peña et al., 2018) were conducted to assess students’ semantics and morphosyntax proficiency. The semantics subtest assessed children’s semantic breadth and depth, including analogies, characteristic properties, categorization, functions, linguistic concepts, and similarities and differences in both English and Spanish. The morphosyntax subtest consisted of cloze questions and sentence repetition tasks in both English and Spanish to assess various simple and complex morphosyntax forms. English semantics raw score, English morphosyntax raw score, Spanish semantics raw score, and Spanish morphosyntax raw score were calculated for both Years 1 and 2.

#### Narrative production ability (Assessed at T1 and T2).

Children’s narrative production was assessed using two wordless storybooks in English and Spanish. Wordless picture books were selected to maximize children’s oral storytelling skills (Paris & Paris, 2003). The storybooks used for assessment were the frog stories, Frog on His Own (Mayer, 1973), One Frog Too Many (Mayer, 1975), Frog Goes to Dinner (Mayer, 1974), and Frog, Where Are You (Mayer, 1969). These books have been widely used by others to examine narrative structure (Norbury & Bishop, 2003; Rojas & Iglesias, 2013; Tompkins et al., 2013). The examiner first modeled a narrative using a wordless book to the child. The model narrative was scripted following Miller and Iglesias (2008). The story script was a story that included a complete episode (e.g., setting, initiating event, attempt, internal response, resolution, and reaction). Children retold the story after the model. Then, the children were given another wordless storybook (for assessment; counterbalanced based on year and language) and were instructed to look through every picture and create a story to tell with reference to the book. The examiner asked the children to return to the book’s first page and prompted the children to tell a story after looking at all the pages. Minimal cues were used by the examiners during the story-retell-tell response. Back-channeling cues were utilized during elicitation of the story in order to ensure children completed the story production and that it could reflect children’s ability.

#### Narrative coding

The stories produced by the participants were coded following Bedore et al. (2010) using a three-step procedure. First, children’s narrative production was recorded via Sony digital voice editor version 2.4.04 and transcribed using the Systematic Analysis of Language Transcripts (SALT) (Miller & Iglesias, 2008). Second, two trained research assistants checked the transcriptions and resolved discrepancies. Finally, for the current study, six measurements, including TNU, TNW, NDW, mean length of utterance in words (MLUw); number of main verbs (MV), and SI, were obtained via SALT for Research using the rectangular database function. TNU was defined as the number of complete and intelligible utterances produced. TNW was referred to as the TNW in all utterances produced, while NDW was defined as the total number of unique words produced in all utterances. MV was referred to as the total MV in all utterances. MLUw was computed by the number of words in all utterances divided by the number of utterances. SI was defined by the total number of main and subordinate clauses divided by the total number of c-units to reflect clausal density. One additional measurement, grammaticality, was measured by the count of grammatical utterances divided by the total grammatical and ungrammatical utterances, after excluding utterances with code-switched elements or a dislocated subject. Thus, 11% of utterances during English storytelling and 12.5% of utterances during Spanish storytelling were excluded from analysis.

#### Data analysis

To address the first research question, descriptive statistics of microstructure derived from narrative production were analyzed. Exploratory factor analysis (EFA) was conducted to examine the factor structure of narrative production. Comparison between factor structures by (i) language and (ii) grade. Based on the results, Bartlett factor scores were generated for (i) productivity of English narrative production, (ii) complexity of English narrative production, (iii) productivity of Spanish narrative production, and (iv) complexity of Spanish narrative production at both Year 1 and Year 2.

**Figure 1.**
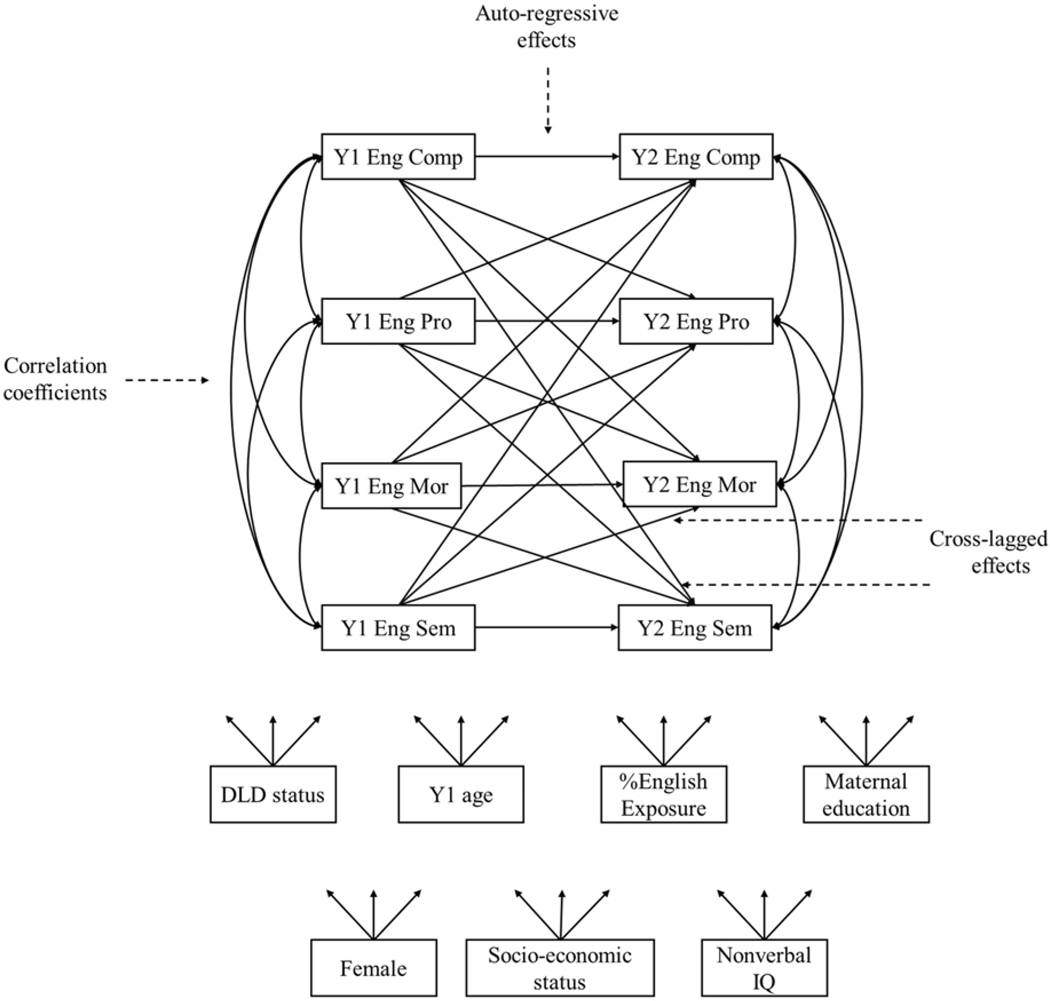
Example of a cross-lagged model.

For the second research question, descriptive statistics and correlation analyses were first performed between bilingual oral language performance and narrative production across Years 1 and 2. Two cross-lagged models were run for English and Spanish separately to examine the longitudinal relationship between language domains and narrative production. Figure 1 shows an example of cross-lagged model. Cross-lagged analysis was selected because it allows examination of (1) autoregressive effects: The effect of a construct on itself at a subsequent time point; and (2) cross-lagged effects: The effect of one construct on another construct at a subsequent time point (Selig & Little, 2012). One potential limitation of the cross-lagged model is that the analysis does not impose explicit theories of developmental patterns (Selig & Little, 2012). However, the current study aimed to investigate longitudinal relationship between language domains and narrative production, and no explicit hypotheses were made based on theory regarding the changes of these constructs. Thus, cross-lagged models fit the study’s purposes in developing an argument for potential causal effects. Cross-lag analyses were conducted using Mplus 8.4 (Muthén & Muthén, 2017) with the maximum likelihood estimator.

Within each language, there were eight variables in the cross-lagged model for Spanish and English, respectively. These variables were semantics, morphosyntax, narrative productivity, and complexity for two time points (Y1 & Y2). For the cross-lagged model, correlation coefficients across clusters within time, auto-regressive effects, and cross-lagged effects were included. Meanwhile, to control for the effects of students’ demographic background on changes in performance, children’s DLD status, gender, age, language exposure, nonverbal IQ, socioeconomic status, and maternal education were controlled at Y1. These control variables were also correlated with Year 1 language skills. To examine the within-language relationship, statistics of auto-regressive effects and cross-lagged effects of Spanish and English were reported.

Another cross-lag model was performed to examine the cross-language longitudinal relationship of narrative production. These variables included narrative productivity and complexity of both Spanish and English for two time points (Y1 & Y2). For the cross-lagged model, correlation coefficients across clusters within time, auto-regressive effects, and cross-lagged effects were included. Meanwhile, to control for the effects of students’ demographic background on changes in performance, children’s DLD status, gender, age, language exposure, nonverbal IQ, socioeconomic status, and maternal education were controlled at Y1. Statistics of auto-regressive effects and cross-lagged effects were reported.

## Results

### Research question 1: factor structure of narrative measurement

Table 2 shows the descriptive statistics of English and Spanish narrative production. We conducted six EFAs that were focused on (i) English narrative production at T1, (ii) English narrative production at T2, (iii) Spanish narrative production at T1, and (iv) Spanish narrative production at T2 with the seven microstructural narrative measurements described in Section 3.3. Bartlett’s sphericity test was significant for all comparisons, *χ^2^*(21) = 1194.36 to 1585.10, *ps* < .001, indicating that the intercorrelation matrix had sufficient common variance for analysis. Using a Promax rotation, eigenvalues larger than 1, and factor loadings higher than .4 as the criteria, a consistent two-factor structure was extracted in all six analyses, which explained 75.17% to 84.15% of the total variance. Table 3 summarizes the factor loading results of the EFA, showing that the first factor consisted of TNU, TNW, NDW, and MV, while the second factor consisted of MLUw, grammaticality, and SI. Based on the measurements within each factor, the first factor was named productivity, while the second factor was named complexity. The two-factor scores (using a Bartlett factor score) in each factor analysis had significantly weak to moderate correlations (*rs* = .26 − .50, *ps* < .01), indicating that the two factors shared similarity, yet captured some distinctive features of narrative production.

Thus, a two-factor structure (productivity and complexity) for micro-level narrative measurements in L1 and L2 of bilingual children was supported. The two-factor model showed stable and significant differences in productivity and complexity. This led to the conclusion that these two factors are indeed separate structures, despite having some commonalities in variance as shown by the correlations between factors. This aligned with the framework by Hughes et al. (1997). The stability of the factor structure suggested the bi-dimensional structure of micro-level narrative measurement for further investigation in research question 2.

#### Research question 2: changes in narrative production

*Oral* language proficiency changes across T1 and T2

Table 2 shows oral language proficiency and narrative production at T1 and T2. The skewness and kurtosis (as shown in Table 2) were regarded as acceptable samples for further statistical analysis suggested by Brown (2015).

#### Cross-lag models on narrative production

**Figure 2.**
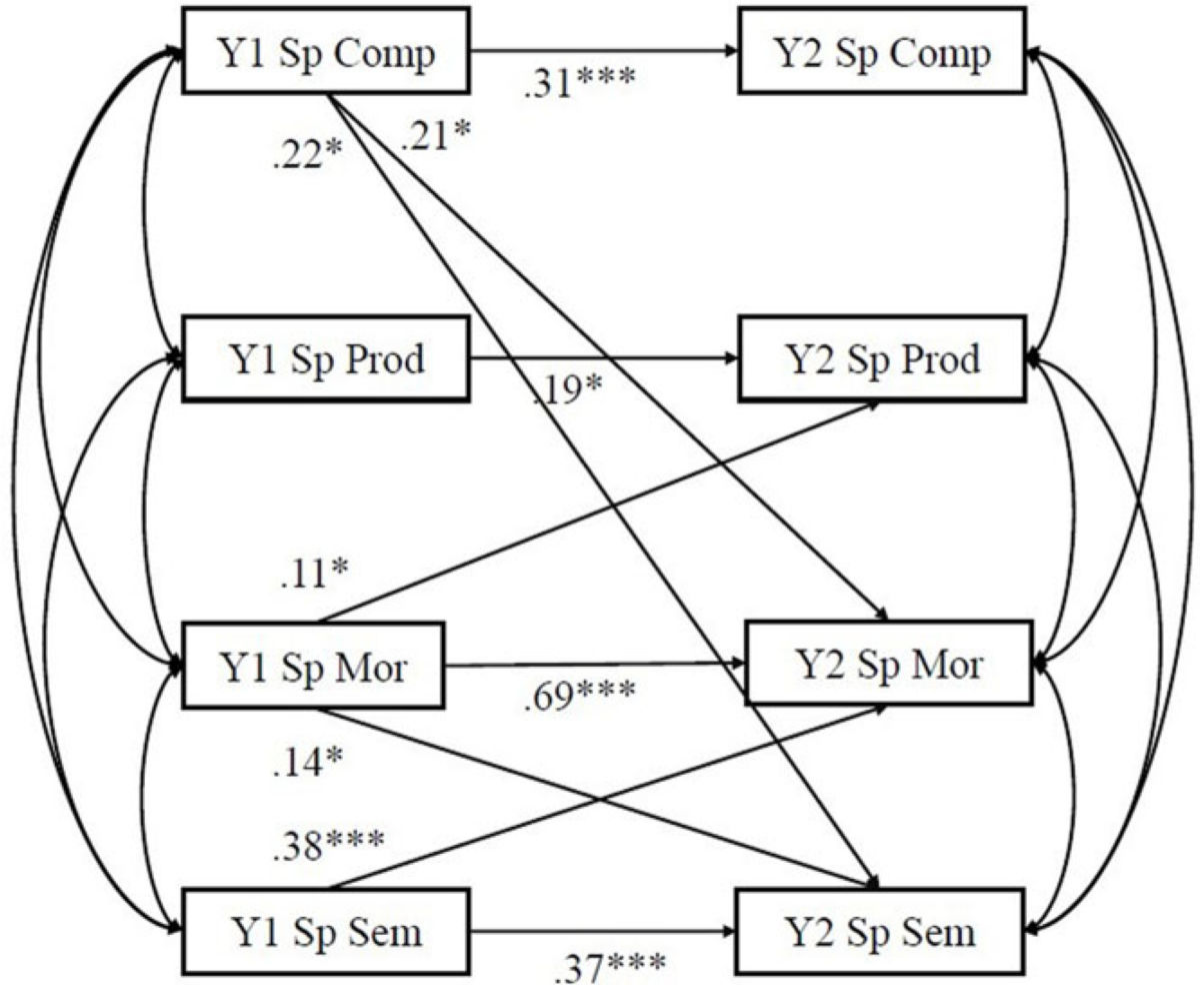
Cross-lagged model for within-language transfer for Spanish. *Note.* DLD status, Year 1 age, gender, percentage of English exposure, nonverbal IQ, socioeconomic status, and maternal education were controlled. Only significant paths were shown. Y1 = Year 1, Y2 = Year 2, Sp = Spanish, sem = semantics, mor = morphosyntax, prod = productivity, comp = complexity. *p < .05, **p < .01, ***p < .001.

**Figure 3.**
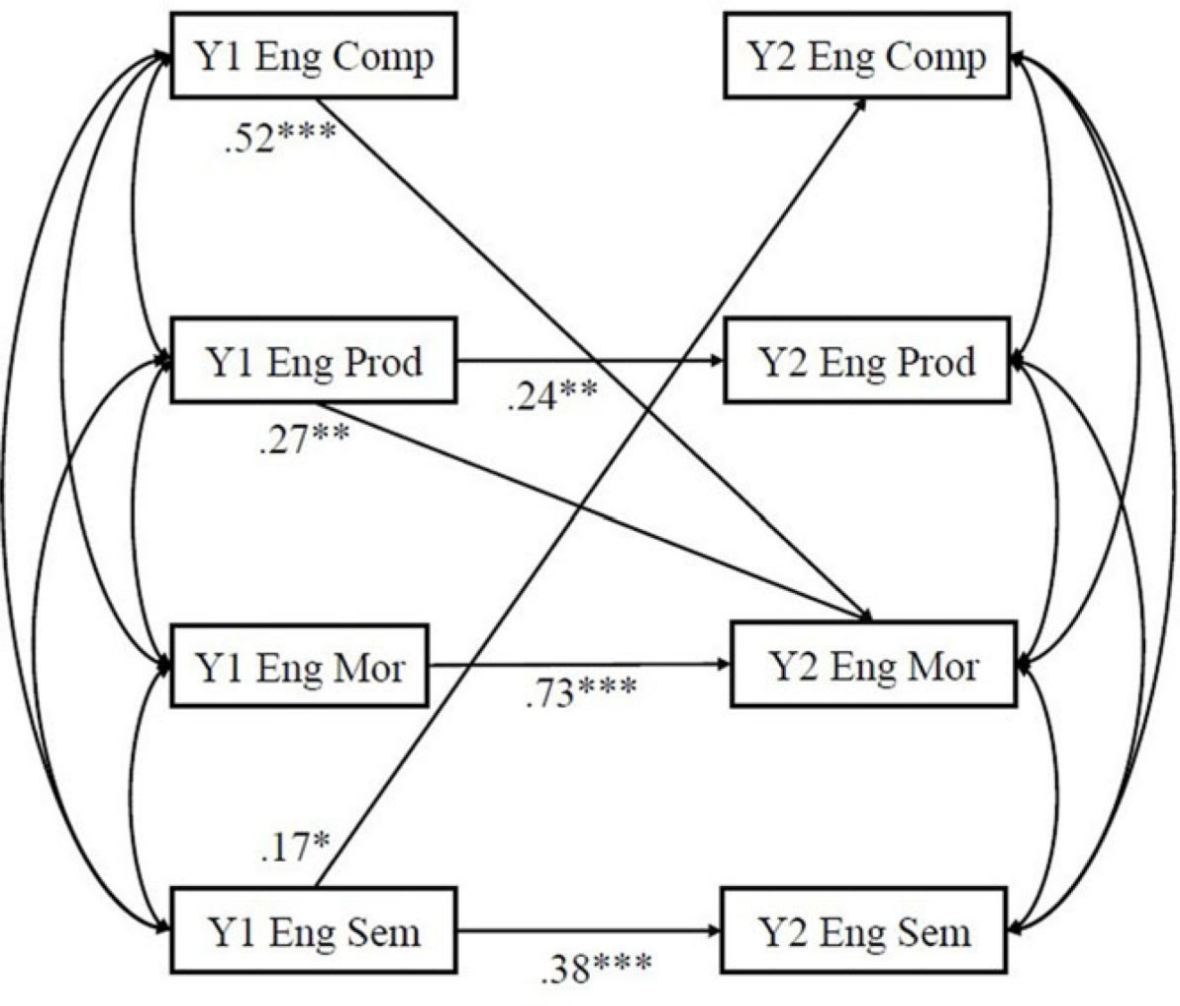
Cross-lagged model for within-language transfer for English. *Note.* DLD status, Year 1 age, gender, percentage of English exposure, nonverbal IQ, socioeconomic status, and maternal education were controlled. Only significant paths were shown. Y1 = Year 1, Y2 = Year 2, Eng = English, sem = semantics, mor = morphosyntax, prod = productivity, comp = complexity. *p < .05, **p < .01, ***p < .001.

Table 4 presents the correlation between variables. Cross-lagged models of within-language relationship between language domains and narrative production of Spanish and English are presented in Figures 2 and 3, respectively. The coefficients of the cross-lagged models are shown in Table 5. All auto-regressive effects for Spanish and English were statistically significant (*β*s ranged from .19 – .73; *ps* < .05) except for complexity of English narrative (*β* = .04, *p* = .62). Auto-regressive coefficients of Spanish and English morphosyntax were large (*β* = .69*, p* < .001*; β* = .73, *p* < .001, respectively), whereas the other auto-regressive effects were of small or medium ranging from .19 to .38 (*ps* < .05).

Different patterns of cross-lag effects were observed in Spanish and English. In Spanish, the cross-lagged effects of Year 1 semantics predicting Year 2 morphosyntax (*β* = .28, *p* < .001), Year 1 morphosyntax predicting Year 2 semantics (*β* = .14, *p* = .03) and Year 2 production of narrative (*β* = .11, *p* = .03), Year 1 complexity of narrative predicting Year 2 semantics (*β* = .22, *p* = .03) and Year 2 morphosyntax (*β* = .21, *p* = .04) were statistically significant. Other cross-lagged relations in the cross-lagged model were not statistically significant.

In English, the cross-lagged effects of Year 1 semantics predicting Year 2 complexity of narrative (*β* = .17, *p* = .01), Year 1 productivity of narrative predicting Year 2 morphosyntax (*β* = .27, *p* = .009), and Year 1 complexity of narrative predicting Year 2 morphosyntax (*β* = .52, *p* < .001) were statistically significant. Other cross-lagged relations in the cross-lagged model were not statistically significant.

For language exposure, after accounting for performance at Year 1, age, gender, maternal education, socioeconomic status, nonverbal IQ and DLD status, the percentage of English exposure negatively predicted all Year 2 Spanish measures (semantics: *β* = −.25, *p* < .001; morphosyntax: *β* = −.23, *p* = .002; productivity of narrative: *β* = −.28, *p* < .001; complexity of narrative: *β* = −.21, *p* = .007). However, the percentage of English exposure only positively significantly predicted Year 2 morphosyntax (*β* = .42, *p* < .001) but not for other measures.

#### Cross-lag models on bilingual narrative production

**Figure 4.**
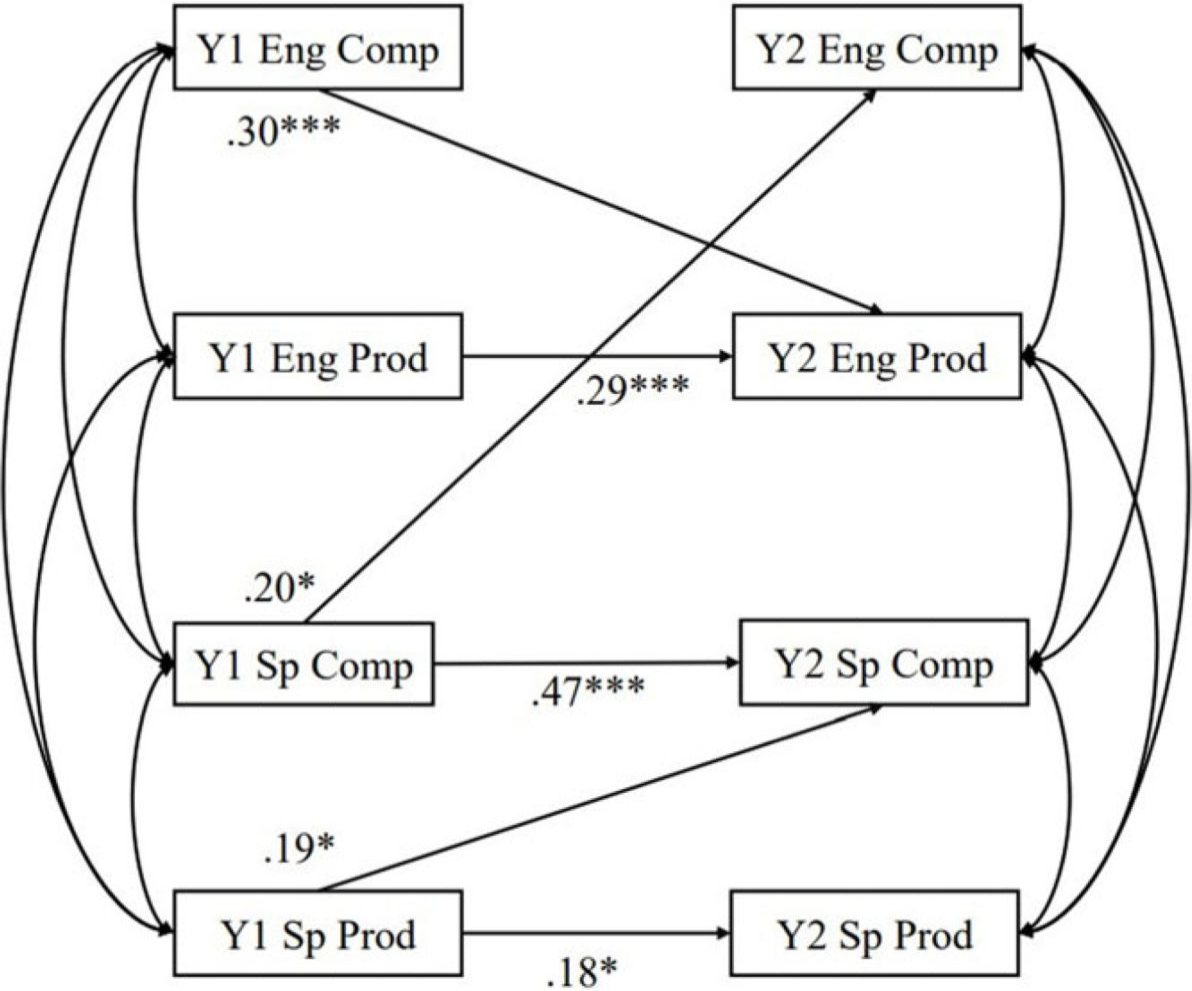
Cross-lagged model for cross-language narrative transfer. *Note.* DLD status, Year 1 age, gender, percentage of English exposure, nonverbal IQ, socioeconomic status, and maternal education were controlled. Only significant paths were shown. Y1 = Year 1, Y2 = Year 2, Sp = Spanish, Eng = English, prod = productivity, comp = complexity. *p < .05, **p < .01, ***p < .001.

Cross-lagged models of within-language relationship between narrative production of Spanish and English are presented in Figure 4. The coefficients of the cross-lagged model are shown in Table 6. All auto-regressive effects for bilingual narrative production were statistically significant (*β*s ranged from .18 – .47; *ps* < .05) except for complexity of English narrative (*β* = .08, *p* = .33). For cross-lagged effect, Year 1 productivity of Spanish narrative predicting Year 2 complexity of Spanish narrative (*β* = .19, *p* = .04), Year 1 complexity of Spanish narrative predicting Year 2 complexity of English narrative (*β* = .20, *p* = .03), and Year 1 complexity of English narrative predicting Year 2 productivity of English narrative (*β* = .30, *p* < .001) were statistically significant. Other cross-lagged relations in the cross-lagged model were not statistically significant.

For language exposure, after accounting for performance at Year 1, age, gender, maternal education, socioeconomic status, nonverbal IQ, and DLD status, the percentage of English exposure negatively predicted Year 2 Spanish narrative measures (productivity: *β* = −.28, *p* < .001; complexity: *β* = −.21, *p* = .003) but was not statistically significant for Year 2 English narrative measures.

## Discussion

We examined 143 Spanish-English bilingual children longitudinally from kindergarten to grade one on language exposure, oral language proficiency, and narrative production. Results indicated that narrative production could be analyzed with a two-factor structure across languages and time. Cross-lag analyses demonstrated that English exposure only negatively predicted change in Spanish narrative production but not for English narrative production. Furthermore, narrative production in Year 1 contributed to language domains (i.e., semantics and morphosyntax) in Year 2 in Spanish and English.

### Stability of two-factor narrative structure model

Factor analyses were conducted to observe how the micro-level narrative measurements could be grouped into similar constructs. The TNU, TNW, number of different words, and MV were loaded onto the construct of productivity, whereas the MLUw, SI, and grammaticality were loaded onto the complexity construct. This suggests that early school-age bilingual narrative production can be analyzed with the same factor structure.

Considering the structure of an individual’s narrative production offers useful insights into their linguistic repertoire and skills that may be difficult to access through a more constrained standardized battery (Fiestas & Peña, 2004). Narrative production is often considered to be longer, more sophisticated, descriptive, and therefore informative than measures that require an individual to give a shorter oral answer. These structures gathered from the participants’ utterances offer in-depth insights into linguistic knowledge that is based on the individual’s ability that is often lost or overlooked in shorter, elicit-response tasks and allow us to group microstructures for better understanding of the underlying processes within narrative production.

The productivity factor encompasses multiple tasks that can be used to consider one’s ability to produce longer utterances through various facets of production (Hughes et al., 1997). The complexity factor includes tasks that consider the depth of the linguistic structures being used in a way that does not focus too much on one particular skill, but rather a comprehensive range of syntactically-focused skills (Hughes et al., 1997). Defining these structures is crucial to understanding language production in linguistically diverse populations. By understanding the factor structure of narrative measurement, researchers and clinical professionals can evaluate the dimensionality of lexical and morphosyntax ability of young bilingual children through less constrained, more naturalistic, or open-ended manners such as storytelling and narrative-based tasks.

### Role of language exposure on bilingual narrative change

Interestingly, cross-lag modeling results showed English language exposure only had a significant prediction of Spanish narrative production but not for English narrative production across three cross-lagged models, when controlling for Year 1 performance and other control variables. These findings were not consistent with the notion that “a necessary trade-off in relative exposure would push across-language correlations in a negative direction” (Hoff et al., 2018, p.2), suggesting that the increase in English exposure does not promote the positive change in English narrative production. On the other hand, this finding reflects prior research that suggested increasing L2 exposure may not be the source of improving L2 narrative for preschoolers and school-age bilingual children (Haman et al., 2017; Hao et al., 2019).

In addition, language exposure was associated with Spanish narrative and oral language domain, suggesting the importance of home language use in early school age, even after formal English instruction at school.

### Role of cross-language transfer in narrative

Strikingly, the cross-lag model of bilingual narrative found a positive prediction from complexity of Spanish to complexity of English. This suggested that L2 narrative development could be explained by the significant cross-language prediction from complexity of Spanish narrative to complexity of English narrative and within-language prediction from English semantics to complexity of English narrative. Thus, bilingual children can use L1 narrative and L2 language domain skills to support the development of L2 narrative. Specifically, complexity of narrative included measurements that related to the production of longer sentences, including MLU and SI. The use of long sentences in L1 narrative with sophisticated L2 semantics skills can facilitate the development of L2 narrative. Given the increasing English demand in the school environment, bilingual students can utilize the language knowledge in Spanish, for example, use of complex sentences in storytelling, to support English language development to fulfill academic needs. Here, the participants in the current study were early school-age, the English instruction at school had likely provided opportunities for bilingual children to acquire morphosyntax and complex syntactic structures for producing a complex narrative. This finding was consistent with the theory of language interdependence (Cummins, 1979) and with prior literature on bilingual narrative transfer (Uccelli & Páez, 2007), suggesting proficiency in L1 can transfer to L2 and provided evidence of specific skill transfer.

### Role of bilingual within-language transfer

In both languages, there was a clear pattern that narrative production in Year 1 weakly to moderately predicted the performance of semantics and morphosyntax in Year 2. These findings suggest that narrative production can be generalized to basic language domains. These results align with the DST, suggesting the *initial state* is important for subsequent development via the *butterfly effect* (de Bot et al., 2007). This means the initial condition at time 1 can affect the development of other neighboring skills at time 2. In the current study, the complexity of narrative production in both languages, which focused on the production of longer, complex, and accurate utterances in narrative context, was shown to predict semantic and morphosyntax performance one year later. This indicated that when children were able to produce complex utterances in a cognitive-demanding yet contextualized task, this can facilitate the generalization to the morphosyntax and semantic performance in both languages. The use of longer, complex, and accurate utterances can provide chances for children to practice complex morphosyntax structures, such as subjunctive and relative clauses in Spanish, and prepositional phrases and relative clauses in English. In addition, with higher productivity and complexity of narrative, children were able to use more different and specific words to describe complex semantic relationships.

### Implications

The stable and identical two-factor structures across languages suggest researchers and educators select corresponding measurements to represent narrative productivity and complexity. In addition, given the importance and cross-language transfer of L1 semantics skills, clinical professionals can consider focusing on the L1 semantic network building and also evaluate the possible generalization across languages and domains. Researchers can also consider this result when they are selecting the generalization outcome of interventions.

These results also have important implications for educators of bilingual students. As shown in the various models and prior literature, the importance of Spanish exposure is critical when considering the growth of Spanish during this stage of development. Educators should work to create opportunities for their students to practice using narrative structures in their home language, as this will help students form well-rounded skills in both target languages to create a wealth of linguistic knowledge. This emphasizes the importance of linguistically rich and diverse interactions in the classroom in order to holistically improve bilingual students’ language learning experience.

### Limitations and future research

Despite the current research providing preliminary evidence on cross-language and within-language transfer in bilingual children, it has two major limitations. First, the current study only focuses on the micro-structure of narrative. While microstructure focuses on word use and morphosyntax at utterance level, there are other aspects of narrative production that reflect language development. Marco structure, such as story grammar and internal state terms, is also another common evaluation aspect for narrative development. Prosody and cohesion are also other aspects of narrative assessment. Another limitation of the current study is the relatively small sample size of the DLD group (*n* = 17). The current study did not examine the differences between typically developing children and children with DLD on the microstructure factors of bilingual narrative production and the change patterns between language domains and narrative production.

Furthermore, given the fact that English language exposure increases during school age (Albudoor et al., 2024; Bedore et al., 2016), future research can consider replicating the experiment at a later school age to investigate the directionality and strength of the within- and cross-language transfer. This may inform Dynamic Systems Theory on how language environment and social factors affect bilingual development.

## Figures and Tables

**Table 1.** Demographics of the participants

Participant Characteristic	*n*	%	*M*	*SD*	Min	Max
Age in months at T1			69.68	4.20	61	79
Age in months at T2			81.96	4.85	73	91
Ethnicity						
Hispanic or Latino	147	94.83				
African American	1	.65				
Others/ Unknown	7	4.52				
Gender						
Female	73	47.10				
Male	80	51.61				
Not provided	2	1.29				
Free/Reduced lunch	120	77.42				
Nonverbal intelligence			97.83	11.91	72	129
Socio-economic status			24.51	10.85	3	66
Maternal education			2.55	1.49	1	6
Percentage of English exposure at T1			58.48	29.37	11.76	100
Percentage of English exposure at T2			64.19	30.82	7.50	100

*Note*. Maternal education: 1 = less than seventh-grade education, 2 = ninth-grade education, 3 = partial high school, 4 = high school, graduate, 5 = partial college or specialized training, 6 = college degree, and 7 = graduate degree. Socio-economic status is calculated by the Hollingshead Four-Factor Index (Hollingshead, 1975).

**Table 2.** Descriptive statistics

	Spanish						English					
	Year 1			Year 2			Year 1			Year 2		
Variable	*M (SD)*	Skewness	Kurtosis	*M (SD)*	Skewness	Kurtosis	*M (SD)*	Skewness	Kurtosis	*M (SD)*	Skewness	Kurtosis
*Oral Language-Domain Proficiency*												

Semantics	24.85 (7.58)	−.43	−.24	31.80 (6.65)	−1.16	2.10	22.27 (7.27)	.11	.08	31.90 (6.19)	−.25	.55

Morphosyntax	43.33 (16.07)	−.59	−.28	38.57 (15.17)	−.41	−.58	24.29 (15.37)	.61	−.56	50.48 (15.29)	−.81	−.03

*Oral Narrative*												

Productivity	−.26 (1.04)	1.26	4.36	.26 (.89)	.54	2.14	−.31 (.98)	.83	1.64	.31 (.92)	−.01	.54

Complexity	−.21 (1.09)	−1.40	4.20	.21 (.86)	−1.50	4.36	−.22 (1.19)	−.94	2.49	.22 (.70)	.02	.70

**Table 3.** Factor loadings of the rotated component matrix on oral narrative

	(a) English at T1			(b) English at T2			(c) Spanish at T1			(d) Spanish at T2		
Component	Prod		Comp	Prod		Comp	Prod		Comp	Prod		Comp
TNU	1.10			1.05			1.07			1.07		

TNW	.93			.99			.93			.98		

NDW	.75			.86			.88			.89		

MV	.90			.98			.96			.95		

MLUw			.83			.69			.88			.60

Grammaticality			.78			.77			.41			.64

SI			.91			.85			.94			.86

Eigenvalues	4.47		1.42	4.41		1.30	4.27		1.37	4.42		1.04

% of variance	63.92		20.24	63.00		18.50	60.96		19.63	63.14		14.89

Cumulative % of variance		84.15			81.50			80.59			78.02	

Correlation between factors			.49***			.42***			.45***			.50***

*Note:* T1 = Time 1, T2 = Time 2, Prod = productivity, Comp = complexity, TNU = total number of utterances, TNW = total number of words, NDW = number of different words, MV = number of main verbs, MLUw = mean length of utterance in words, SI = subordination index. Extraction method: principal component factoring; rotation method: promax rotation with Kaiser normalization, rotation converged in 3 iterations for all analyses.

** *p* < .01,

*** *p* < .001.

**Table 4.** Correlation between bilingual oral language-domain proficiency and factor score of bilingual oral narrative

	1	2	3	4	5	6	7	8	9	10	11	12	13	14	15
1. Y1 Sp sem	—														
2. Y1 Sp mor	.67***	—													
3. Y1 Eng sem	.44***	.29**	—												
4. Y1 Eng mor	.17*	.22**	.63***	—											
5. Y1 Sp prod	.20*	.19*	−.04	−.15	—										
6. Y1 Sp comp	.45***	.55***	.11	.06	.43***	—									
7. Y1 Eng prod	.03	−.01	.29***	.38***	.17*	−.02	—								
8. Y1 Eng comp	.19*	.22**	.49***	.59***	.13	.36***	.47***	—							
9. Y2 Sp sem	.64***	.64***	.33***	.13	.28**	.49***	−.07	.12	—						
10. Y2 Sp mor	.10	.21*	.51***	.83***	−.15	.08	.40***	.61***	.12	—					
11. Y2 Eng sem	.38***	.34***	.63***	.56***	.07	.21*	.24**	.40***	.42***	.55***	—				
12. Y2 Eng mor	.62***	.84***	.15	.09	.26**	.53***	−.08	.12	.69***	.11	.31***	—			
13. Y2 Sp prod	.20*	.16	−.03	−.10	.34***	.28**	.10	.00	.41***	−.08	.17*	.29***	—		
14. Y2 Sp comp	.32***	.54***	.02	.00	.34***	.52***	.12	.18*	.51***	.07	.19*	.59***	.49***	—	
15. Y2 Eng prod	.07	.04	.42***	.43***	.07	.13	.37***	.37***	.21*	.52***	.47***	.06	.29**	.14	—
16. Y2 Eng comp	.23**	.16	.41***	.20*	.14	.17*	.18*	.23**	.24**	.23**	.32***	.13	.18*	.09	.34***

*Note:* Y1 = Year 1, Y2 = Year 2, Sp = Spanish, Eng = English, sem = semantics, mor = morphosyntax, prod = productivity, comp = complexity.

* *p* < .05,

** *p* < .01,

*** *p* < .001.

**Table 5.** Coefficients for within-language cross-lagged models

Main Effect			Effects of Control Variables					
	Spanish	English			Sem	Mor	Prod	Comp
*Cross-lagged effects*			*Control Variables*					

Y1 Sem → Y2 Mor	.38***	.14	DLD status	Spanish	−.36***	−.38***	.00	−.25***

Y1 Sem → Y2 Prod	−.02	.08		English	−.30***	−.32***	−.18*	−.25***

Y1 Sem → Y2 Comp	−.14	.17**	Y1 Age	Spanish	.14	.08	.00	.10

Y1 Mor → Y2 Sem	.14*	.11		English	.16*	.26***	.08	.18***

Y1 Mor → Y2 Prod	.11*	.00	%English exposure	Spanish	−.25***	−.23**	−.28***	−.21**

Y1 Mor → Y2 Comp	.10	−.05		English	.12	.42***	.14	.00

Y1 Prod → Y2 Sem	.04	−.12	Material education	Spanish	−.10	−.08	−.04	−.08

Y1 Prod → Y2 Mor	.01	.27**		English	.11	.07	.10	.15

Y1 Prod → Y2 Comp	.17	.04	Female	Spanish	.02	.04	−.04	.03

Y1 Comp → Y2 Sem	.22*	.05		English	−.01	−.04	−.07	.09

Y1 Comp → Y2 Mor	.21*	.52***	Socioeconomic status	Spanish	−.11	−.15*	.00	−.05

Y1 Comp → Y2 Prod	−.03	.06		English	.20**	.06	.07	.03

*Auto-regressive effects*			Nonverbal IQ	Spanish	.11	.06	.10	.06

Y1 Sem → Y2 Sem	.38***	.38***		English	.23**	.05	.07	−.02

Y1 Mor → Y2 Mor	.69***	.73***						

Y1 Prod → Y2 Prod	.19*	.24**						

Y1 Comp → Y2 Comp	.31***	.04						

*Note*: All path coefficients are standardized results. Y1 = year 1, Y2 = year 2, sem = semantics, mor = morphosyntax, prod = productivity, comp = complexity.

* *p* < .05;

** *p* < .01;

*** *p* < .001.

**Table 6.** Coefficients for cross-lagged models for bilingual narrative production

			Effects of Control Variables			
Main Effect			Sp Prod	Sp Comp	Eng Prod	Eng Comp
*Cross-lagged effects*		*Control Variables*				

Y1 Sp Prod → Y2 Sp Comp	.19*	DLD status	.00	−.25**	−.18*	−.24**

Y1 Sp Prod → Y2 Eng Prod	−.01	Y1 Age	−.01	.10	.08	.18*

Y1 Sp Prod → Y2 Eng Comp	.07	%English exposure	−.28***	−.21**	.14	.00

Y1 Sp Comp → Y2 Sp Prod	−.03	Material education	.08	−.05	.07	.03

Y1 Sp Comp → Y2 Eng Prod	.06	Female	−.04	.03	−.07	.09

Y1 Sp Comp → Y2 Eng Comp	.20*	Socioeconomic status	.00	−.05	.07	.03

Y1 Eng Prod → Y2 Sp Prod	.00	Nonverbal IQ	.10	.06	.07	−.02

Y1 Eng Prod → Y2 Sp Comp	.08					

Y1 Eng Prod → Y2 Eng Comp	.04					

Y1 Eng Comp → Y2 Sp Eng Prod	−.14					

Y1 Eng Comp → Y2 Sp Comp	.06					

Y1 Eng Comp → Y2 Eng Prod	.30***					

* Auto-regressive effects*						

Y1 Sp Prod → Y2 Sp Prod	18*					

Y1 Sp Comp → Y2 Sp Comp	47***					

Y1 Eng Prod → Y2 Eng Prod	29***					

Y1 Eng Comp → Y2 Eng Comp	.08					

*Note*: All path coefficients are standardized results. Y1 = year 1, Y2 = year 2, Sp = Spanish, Eng = English, prod = productivity, comp = complexity.

* *p* < .05;

** *p* < .01;

*** *p* < .001.
